# Public Perceptions of Service Dogs, Emotional Support Dogs, and Therapy Dogs

**DOI:** 10.3390/ijerph14060642

**Published:** 2017-06-15

**Authors:** Regina Schoenfeld-Tacher, Peter Hellyer, Louana Cheung, Lori Kogan

**Affiliations:** 1 Department of Molecular and Biomedical Sciences, College of Veterinary Medicine, North Carolina State University, Raleigh, NC 27607-7302, USA; 2 Department of Clinical Sciences, College of Veterinary Medicine and Biomedical Sciences, Colorado State University, Fort Collins, CO 80523, USA; Peter.Hellyer@colostate.edu (P.H.); lori.kogan@colostate.edu (L.K.); 3 College of Veterinary Medicine and Biomedical Sciences, Colorado State University, Fort Collins, CO 80523, USA; louana.cheung@colostate.edu

**Keywords:** public perception, service dogs, therapy dogs, emotional support animals, assistance animals, Americans with Disabilities Act, Housing and Urban Development Regulations

## Abstract

As service dogs, emotional support dogs, and therapy dogs have become more prevalent in the USA, so too has the controversy surrounding their legitimacy. Yet, there is a lack of objective data regarding the public’s understanding of the role played by each of these types of animals, as well as their perceptions regarding the legitimacy of their integration. An anonymous, online survey was distributed to examine the perceptions of US adults who do not own any type of assistance animal. A total of 505 individuals responded to the online survey, yielding 284 usable responses. Results suggest widespread misconceptions about definitions, rules, regulations, and rights associated with each type of assistance dog. In general, service dogs are more likely to be perceived as helping with a legitimate need, and their access to public spaces is viewed favorably. While there are some concerns about the legitimacy and necessary access rights for emotional support dogs, members of the public correctly identified the roles and rights of therapy dogs. Despite the media’s focus on abuses and false representation of these dogs, most participants reported feeling the majority of people are not taking advantage of the system.

## 1. Introduction

### 1.1. Definitions of Assistance Animals

The number and types of helping roles fulfilled by assistance animals, most often dogs, have been growing rapidly [1]. As a result, there is considerable confusion over the meaning of individual terms used to designate the function and role of service animals, assistance animals, therapy animals, and emotional support animals [2]. Further complicating the issue is the fact that different organizations and statutes use different terms to designate the rights and privileges of various types of assistance animals. In the simplest of terms, assistance animals can be viewed as an umbrella label, with sub-categories including, but not limited to, service animals, therapy animals, and emotional support animals.

A service animal is defined as an animal that provides assistance related to a person’s disability, and enjoys broad access to public locations under the Americans with Disabilities Act, Title II and Title III [2,3,4]. The ADA regulations specify that only dogs, or in specific cases, miniature horses, may work as service animals. Service animals have an advanced level of training relative to typical dogs. Nationally-recognized certification programs are available, but not mandated, for service animals. Psychiatric service dogs are a recognized sub-category of service animals. They assist people who suffer from major depression, bipolar disorder, anxiety, panic attacks, PTSD, OCD, and schizophrenia. These dogs are trained to carry out specific tasks to help an individual cope with his/her disability [5].

In contrast, an emotional support animal (ESA) also provides assistance related to a psychological disability, but is not required to have any specific training. Any species of animal may act as an emotional support animal. ESAs are “owned” animals/pets. There are no certification or registration standards for ESAs, and their access to public locations is more limited. However, federal regulations allow them to reside in both public and private housing. These are codified in Title VIII of the Civil Rights Act of 1968, known as the Fair Housing Act (FHA) [6,7]. Finally, a therapy animal may or may not provide assistance related to a disability. There are no specific laws governing the species that may work as therapy animals. These animals can have a varying level of training, relative to what might be expected of a typical pet. This type of animal assists professionals, such as counselors, social workers, and physical therapists, in performing their work with clients. However, therapy dogs are not guaranteed access to public facilities under any type of statutes [2]. Further complicating this distinction is the possibility that a therapist or service professional might have an ESA for their personal needs and also register the same dog as a therapy dog for use in their work with clients.

### 1.2. Access and Legal Statutes

The Americans with Disabilities Act (ADA), updated in 2010, defines service animals as “dogs that are individually trained to do work or perform tasks for people with disabilities” [8]. The explanation immediately following this definition expands upon the roles of service dogs, making it clear that in addition to performing physical tasks, animals that provide assistance for individuals with psychiatric disabilities, such as calming a person with PTSD during an anxiety attack, also qualify as service dogs. Taken together, ADA Title II and Title III [3,4] stipulate that state and local governments, businesses, and nonprofit organizations that serve the general public must allow service dogs to access all areas open to the public. This ensures the dogs’ abilities to assist their handlers in order to participate in activities of daily living, study, work, etc. It is very important to note that while the ADA definition encompasses dogs (and miniature horses in specific cases) that provide service to individuals with psychiatric disabilities, it specifically excludes “dogs whose sole function is to provide comfort or emotional support” [8].

Emotional support animals are defined as an animal of any species that provides emotional support and/or therapeutic benefit to an individual with a verifiable mental or psychiatric disability. While there is no nationwide standard for certification or registration of ESAs, many online agencies claim to “register” an animal as an ESA for a fee (e.g., $79.00 at the United States Dog Registry) [9], while an emotional support animal identification kit can be purchased online from a variety of websites (e.g., $39.00 from Service Dog Certifications) [10]. Neither registration nor identification are required, however. Access rights for ESAs are governed by the Fair Housing Act of 1988 [11], along with the amendments specified in FHEO 2013-01 [7]. These regulations define a disabled person as any person who has a physical or mental impairment that substantially limits one or more major life activities and has a record of such impairment, or is regarded as having such an impairment [6,7]. The FHA regulations consider ESAs to be a reasonable accommodation for a disability, analogous to a wheelchair or other physical devices, which help individuals cope with a disability. This distinction is important, as it means that only service animals have broad access rights to public areas under ADA laws. In contrast, the access rights of ESAs are limited to residential dwellings as defined by FHA rules. As an additional note, even though local ordinances may impose bans on certain breeds of dogs, the ADA regulations supersede these bans. Thus, service dogs and assistance animals are not bound by weight, size, or breed restrictions [12], and landlords may not refuse to rent a property to someone with a service dog or ESA, nor charge them an additional deposit for these types of animals.

During air travel, the Air Carrier Access Act (DOT rule Title 14 CFR Part 382) protects the rights of passengers with disabilities [13,14]. This legislation utilizes a broad definition of disability, including any individual who has a physical or mental impairment that substantially limits one or more major life activity. Mental impairment is explained to include any mental or psychological disorder, such as mental retardation, organic brain syndrome, emotional or mental illness, and specific learning disabilities. This regulation encompasses both service dogs and ESAs, stating that “Carriers shall permit dogs and other service animals used by persons with a disability to accompany the persons on a flight”. This definition is followed by a statement outlining the types of acceptable evidence to demonstrate that an animal is a service animal, including: service animal identification cards, other written documentation, presence of harnesses or marking on harnesses, tags, or the credible verbal assurances of the qualified individual with a disability using the animal. There are no Federal regulations governing public access for therapy dogs.

### 1.3. Required Training, Certifications, and Conditions for Removal

The major differences between service dogs and ESAs revolve around their required training. While service dogs must be trained to perform specific tasks for the benefit of an individual with a disability, no such training is required for ESAs. ESAs may accomplish their task by providing companionship or alleviating loneliness. These differences may make it difficult for landlords, business owners, and employers to distinguish between the types of assistance animals. ADA regulations stipulate that the only questions which may legally be asked to establish if an animal is a service dog/miniature horse are (1) “Is this a service animal?” and (2) “What service/skill/task does the animal do?” Questions pertaining to an individual’s disability are specifically forbidden. In contrast, while no special training or certification is required to be designated as an ESA, housing providers are required to evaluate requests for an ESA by following the general principles for all reasonable accommodations. Providers much consider whether the person seeking housing has a disability and whether their need for an assistance animal is related to the disability. If the disability and/or need for the ESA is not apparent, the provider may ask the tenant to provide a letter from a mental health professional, addressing how the ESA alleviates symptoms associated with their disability [7]. Both service dogs and ESAs must be under effective control (usually on a leash) at all times, and follow all state laws and local ordinances. ADA law specifies that premises owners or other responsible personnel may ask that a service dog be removed if it is out of control and the handler does not take effective action to control it, or if it is not housebroken.

### 1.4. Laws Regarding Fraudulent Representation

Given the somewhat overlapping terminology and recent proliferation of service dogs and ESAs, stories abound of people taking advantage of unclear policies [15]. To address the concern of fraud, many states are implementing new laws, most of which focus on service animals. A total of 19 states currently have laws in place regarding fraudulent representation of a service animal [16]. Yet, there is great variation in the breadth and penalties associated with these laws. For example, New Jersey defines fraudulent representation as “fitting a dog with a harness of the type commonly used by blind persons to represent that such dog is a guide dog when not trained as a guide dog (NJ SA 10:5-29.5) [17]. In contrast, Utah takes a much broader view of misrepresentation. In addition to a clause about false representation of an animal as a service animal, the state also considers “knowingly and intentionally representing a material fact to a health care provider for the purpose of obtaining documentation from the health care provider necessary to designate an animal as a service animal” as a class B misdemeanor (U.C.A. 1953 § 62A-5b-106) [18].

Considering the range of operational and legal definitions of assistance animals, considerable variation is expected in the public’s understanding and perceptions of laws regarding service animals. This study was designed to report on US adults’ (who do not own an assistance animal) general understanding of assistance animals (tasks performed and pertinent laws), as well as their perception of the prevalence of fraudulent representation. While it is possible for an animal of any species to work as an emotional support or therapy animal, this paper will solely focus on public perceptions of dogs, in each of these roles.

## 2. Materials and Methods

An online survey was created in Qualtrics to assess perceptions held by members of the United States general public regarding service dogs, emotional support dogs and therapy dogs. The survey was designed, reviewed, and tested by the co-investigators and their colleagues at Colorado State University (CSU) and North Carolina State University (NCSU) after seeking input from representatives from the community who either trained, owned, or interacted with either service, emotional support, or therapy dogs. They were asked to provide feedback on content, navigability, survey questions and choices, and overall questionnaire design. Revisions were made based on this feedback in an ongoing process that involved several reiterations. After this step was completed, the survey was tested by approximately ten individuals for any additional ambiguity and/or potentially missing or inappropriate response options. The survey originated from CSU and received approval from the Institutional Review Board at CSU. The online survey was posted as a human intelligence task to Amazon Mechanical Turk (MTurk). Participants were recruited online, from 21 March 2017 to 4 April 2017. A small payment was offered for completion of the task, and offered to a total of 505 participants. All data were collected anonymously. The survey began by asking participants to rate their ability to define the terms service dog, therapy dog, and emotional support dog. After participants responded to this question, definitions of each type of assistance dog were provided for use in the remainder of the survey.

The next section of the survey asked participants to indicate their perceptions of the value and acceptability of service, emotional support and therapy dogs, followed by a set of questions inviting respondents to estimate the percentage of people misrepresenting a need for a service or emotional support dog. Subsequent sections enquired about the questions that can legally be asked of someone with a dog that appears to be an assistance animal, as well as the types of settings in which these dogs should be allowed. A true/false group of questions then assessed participants’ understanding of the regulations surrounding each type of assistance dog. The next question set examined participants’ ability to discern between service, emotional support and therapy dogs. The last section of the survey contained demographic questions, such as age and educational level. It also asked about respondents’ exposure to assistance animals in general, and whether they owned a service or emotional support dog.

Survey responses were downloaded into SPSS (IBM Corp, Released 2012, IBM SPSS Statistics for Windows, Version 21.0, Armonk, NY, USA) for data analysis. The initial 505 submissions were screened in order to exclude respondents who personally owned an emotional support or service dog. The remaining data sets were screened for quality control, via a set of three questions with required responses. Participants who did not follow instructions for all three screening questions (e.g., enter the word “dog” in this blank) were removed from further analysis, yielding a total of 284 usable responses. This is roughly equivalent to a 56.3% response rate.

Descriptive statistics and frequency distribution (reported in percentages) were performed using commercially-available software Chi-square was used to test for any differences in perceived prevalence based on respondents’ gender, age, educational level, and personal pet ownership on misrepresentation of a need for an assistance dog (service or emotional support). Comparisons were also made between respondents with greater exposure to assistance dogs (owned by friends/family), and those who did not report such experiences. Differences were considered significant when *p* < 0.05.

## 3. Results

The online survey was posted from 21 March 2017 to 4 April 2017 with a total of 505 responses obtained. The data was then screened as described above, resulting in a total of 284 responses. However, the total number of responses for each question varied, since not all participants answered each question, therefore, the total numbers of responses are indicated where necessary.

The gender distribution of participants was approximately equal, with 135 males (47.5%) and 149 females (52.5%) represented in the sample. Most participants (131, 46.1%) were between the ages of 26 and 35 years old, and the vast majority had completed a college degree, with 97 (34.2%) reporting completion of a four-year degree, and an additional 115 (40.5%) indicating they held a graduate degree (Table 1). Slightly more than one-half of the participants (153, 53.9%) reported owning a pet dog, while 108 (38.0%) indicated they had a friend or family member who owned a service dog, and 109 (38.4%) reported having a friend or family member who owned an emotional support dog.

When first asked to rate their ability to define three types of service dogs prior to any definitions given (Table 2), the majority of participants rated themselves as very comfortable or somewhat comfortable in defining service dog, emotional support dog, and therapy dog, with service dog receiving the largest proportion of very comfortable ratings (151, 53.2%).

Once participants had rated their ability to define each type of assistance dog, they were presented with the definitions listed below, and asked to answer the rest of the survey using these definitions.

- Service dog—a dog that is individually trained to do work or perform tasks for the benefit of an individual with a disability, including a physical, sensory, psychiatric, intellectual, or other mental disability.
- Emotional support dog—a dog that provides companionship, relieves loneliness, and can help with depression, anxiety, and certain phobias, but does not have special training to perform tasks that assist people with disabilities.
- Therapy dog—a dog that provides people with therapeutic contact, usually in a clinical setting, to improve their physical, social, emotional, and/or cognitive functioning.

### 3.1. Perceptions of Assistance Dogs

The majority of participants agreed or strongly agreed that it was acceptable for people to have a service dog if it was deemed helpful. Approximately half of the respondents disagreed (67, 23.6%) or strongly disagreed (90, 31.7%) with limiting the privileges granted to emotional support and therapy dogs. After completing these questions, 21.1% of participants were still not confident in their understanding of the differences between service dogs, emotional support dogs, and therapy dogs (Table 3).

As seen in Table 4, there was a substantial amount of confusion about the questions that can be legally asked to determine if a dog qualifies as an assistance dog. Most respondents (163, 57.4%) recognized that it is illegal to ask an individual to provide proof of a disability, and a slightly smaller number (138, 48.6%) correctly stated that assistance dog handlers may not be asked to identify their disability. In regards to the dogs themselves, the majority of respondents correctly recognized what is allowable to enquire about the tasks an assistance dog is trained to perform (159, 56.0%). However, the responses were approximately evenly distributed when it came to enquiring if the service dog is required because of a disability. Only 113 (39.8%) of participants correctly identified this question as permissible. Also of concern was the fact that just 81 participants (28.5%) selected the correct response regarding a request to see proof of the dog’s status, such as an identification card or certificate.

Overall, the public seems to agree that assistance dogs should have broad access to residential dwellings, transportation, and educational institutions. Service dogs appeared to have the greatest rate of acceptance, with 172 participants (60.6%) endorsing service dogs’ right to ride in airplane cabins, 170 (59.9%) agreeing they should be allowed to reside in school dormitories, and 163 (57.4%) agreeing that they should be allowed in classroom settings. Only 43 participants (15.1%) felt that landlords should be able to refuse service dogs. The response frequencies for emotional support dogs were slightly less favorable, with less than half of the participants feeling they should be allowed in airplane cabins, dormitories, or classroom settings, but only 54 respondents (19.0%) felt that landlords should be able to exclude emotional support dogs. The responses for therapy dogs were the least favorable regarding access to public spaces. Finally, 10–13% of participants expressed a lack of understanding of what is allowable for all types of assistance dogs (Table 5).

### 3.2. Prior Interactions with Assistance Dogs

Most respondents had minimal prior contact with assistance dogs in public settings. Approximately half (121, 49.6%) of respondents had only minimal exposure to assistance dogs in public venues such as work, school, and airports in the year preceding the study. Fifty-five participants (19.4%) reported no exposure in public settings, with just 88 participants (31.0%) reporting 5 or more sightings or interactions with assistance animals.

### 3.3. Assistance Animal Misrepresentation

The relationship between respondents’ gender and rate of perceived prevalence of assistance animal misrepresentation was explored for both service dogs and emotional support dogs (Table 6). The chi-square statistic was significant (*p* = 0.007), with more women than men reporting lower incidence rates of service dog misrepresentation. A similar pattern (chi-square *p* = 0.025) was found for the misrepresentation of a need for an emotional support dog. There was no relationship between participants’ highest level of education and their perceptions of misrepresentation for either type of assistance animal. Participants’ age was related to their perception of misrepresentation of a need for a service dog (chi-square = 0.001), but not for an emotional support dog (chi-square = 0.063).

The effects of canine companionship, as well as exposure to assistance animals on participants’ perception of misrepresentation was also examined (Table 7). Participants with friends/family who owned a service dog were more likely to suspect misrepresentation of a need for a service dog (chi square < 0.000) or emotional support dog (chi square < 0.000), than those without contextual exposure to service dogs. A similar trend was found for suspected misrepresentation of need for an emotional support dog. Participants with friends/family who owned a service dog (chi square = 0.029) or emotional support dog (chi square = 0.001) were more suspicious of misrepresentation than those without such exposure.

## 4. Discussion

As assistance animals become more prevalent in society, there appears to be a parallel increase in the frequency of allegations of misrepresentation or fraudulent representation of animals as assistance animals. However, no objective studies have been conducted to characterize the public’s understanding of the varying roles and rights of assistance animals, the laws governing their access to public spaces, or their general perceptions.

The inconsistency in terminology used to describe assistance animals, as well as the lack of standardized certification requirements for these animals and overlapping sets of legal regulations make it difficult for the public to determine an animal’s role or job [2]. This confusion paves the way for misrepresentation of pets as assistance animals in order to gain illegitimate benefits, thereby increasing skepticism and scrutiny of legitimate assistance animals. Even though 19 states have passed laws against fraudulent representation of assistance animals [16], public confusion and lack of a standardized certification process makes these laws all but unenforceable from a practical point of view. The goal of this study was to address public perception regarding three types of assistance dogs: service dogs, emotional support dogs, and therapy dogs.

In this study, despite expressing a high level of confidence in defining the roles of service dogs, emotional support dogs, and therapy dogs, participants were still not able to fully apply these definitions and identify the legally-acceptable questions they can ask to identify a true assistance dog. It is interesting to note that a greater percentage of respondents expressed confidence in their ability to define a service dog than an emotional support dog or a therapy dog. This might be related to the longer history of working with assistance animals as guide dogs for the blind [19], as well as the more readily observable tasks performed by a service dog assisting a physically handicapped user. In contrast, emotional support dogs alleviate an invisible disability, making it more challenging for casual observers to understand their role and how they are helping an owner with a mental illness. After reading the definitions of each type of assistance dog, study participants indicated their agreement with a series of statements about the perceived ability of an assistance animal to help users with a legitimate disability. Overall acceptance of assistance animal ownership was high, with the majority of participants agreeing that there is nothing wrong with people having an assistance animal if they feel it is useful. Once again, service dogs generated the highest rate of acceptance. But, it is interesting to note that a higher percentage of respondents strongly agreed with the statement that there “I see nothing wrong with providers using therapy dogs if they think they are helpful” than did with a similar statement regarding the use of emotional support dogs. This might be related to the perceived value/legitimacy of these animals, or to the clearer definition of a therapy dog’s role. Even though service dogs were more broadly accepted, participants still felt that other types of assistance dogs should be given special privileges. However, despite voicing these strong opinions, 21% of participants stated that they were not comfortable describing the differences among assistance animals, thus calling into question the knowledge base for their opinions.

This issue is further complicated by the difficulties associated with identifying a legitimate assistance dog in a public setting. In order to protect the rights of disabled individuals, the ADA limits the questions that may be asked when the type of service being provided by an assistance animal is not obvious. Under these laws, people may only ask if the dog (or miniature horse) is a service animal that is required because of a disability, and what work or task the dog has been trained to perform. Titles II and III of the ADA apply to state/government entities and areas of public accommodation [3,4]. Since access to residential dwellings is governed by the Fair Housing Act, providers are allowed to ask individuals seeking emotional support animals (of any species) as a reasonable accommodation to provide documentation from a health professional. This letter must establish that the individual in question has a disability, and the emotional support animal will provide some type of disability-related assistance or emotional support [7]. While airlines may sometimes request this same type of letter, this type of proof is not required under the Air Carrier Act. Instead, airlines must accept identification cards, other written documentation, the presence of harnesses, tags, or the credible verbal assurances of a qualified individual with a disability using the animal [13,14].

Against this backdrop of overlapping regulations, the broad range of opinions regarding allowed access levels for each type of assistance dog isn’t surprising (Table 5). The majority of participants felt service dogs should be allowed in airplane cabins, school dormitories and classrooms. These perceptions are in line with current laws regarding access to public areas for service dogs. A potential area for concern is that 15.1% of participants felt that landlords should be able to refuse service dogs. This practice is illegal under the FHA [6,7]. The mismatch with public perceptions may be due to a lack of understanding—either of the role of a service dog, or of the overlapping laws governing access to housing.

Public opinion regarding access for emotional support dogs was not as positive, with only 40.1% of respondents agreeing they should be able to ride in airplane cabins. Although this right is protected by the Air Carrier Act [13,14], the lower threshold of proof required for access may have resulted in a greater number of people having a negative perception of emotional support dogs. This could be due to an encounter with a poorly-trained emotional support animal, or one perceived to be fraudulent, in the confines of an airplane cabin. The FHA rules about emotional support animals also apply to residential dwellings, as well as school dormitories, guaranteeing these animals the right to reside with their owners [7]. Thus, it is concerning to see that 19.0% (*n* = 54) of the public feels that property owners should be able to refuse emotional support dogs within their properties, and begs the question of what might be driving this opinion. Moreover, while a sizeable percentage of respondents (46.1%, 131) agreed that emotional support dogs should be allowed to reside in school dormitories, the remaining 53.9% either opposed this accommodation or were unsure as to whether it should be available. While we might surmise why this perception is held (e.g., college students’ lack of time to properly care for assistance animals), the legal system has previously ruled that dormitories are dwellings [20,21]. Public endorsement of emotional support dogs in classrooms was poor, with only 34.5% (98) of respondents supporting their right to be present in classroom settings. This opinion matches current laws, as emotional support animals are not guaranteed the right to be present in classroom settings. Masinter [22,23] explains that even if an ESA is allowed to live in campus housing, an independent determination should be made as to whether the ESA is also needed for classroom access, based solely on the ADA and section 504 of the Rehabilitation Act standards.

Overall, participants’ responses regarding therapy dogs were more closely aligned with existing legislation, which does not grant this type of dog any special access rights. Respondents were more likely to agree that property owners should be able to refuse therapy dogs (22.5%, 64) than other types of assistance animals. This belief matches FHA law, which does not consider therapy dogs to be a reasonable accommodation [6,7] because they are not providing a service to a specific individual. Interestingly, the public was slightly more supportive of therapy dogs being allowed in classroom settings (38.0%, 108) than they were of emotional support dogs, and about equally supportive of therapy dogs riding in airplane cabins (40.5%, 115). Therapy dogs were the least likely to be viewed as appropriate for residing in school dormitories (39.4%, 112). The reasons behind this perception are an interesting area for future research, especially in light of the already-existing processes for evaluation and registration of therapy dogs. We speculate that public perceptions may be based upon the definition of therapy dogs as being part of a treatment team, and the assumption that professionals, such as therapists and counselors do not usually reside in dormitories.

Even though most participants in this study had minimal prior contact with service dogs and emotional support dogs, their overall perception of the legitimacy of these types of assistance dogs was quite high. As seen in Table 6, most respondents felt that there were relatively low incidence rates of misrepresentation. The chi-square comparison for gender found that women reported lower perceived rates of misrepresentation for both service dogs and emotional support dogs than men. This might be because women are, by nature, more trusting, as research has found that increased levels of testosterone correlate with increased levels of suspicion [24] and decreased interpersonal trust [25]. Furthermore, women are more likely to regain trust after repeated transgressions [26], which could explain why even multiple exposures to animals clearly misrepresented as assistance dogs would make less of an impression on female respondents.

Participants’ age was related to their perception of fraudulent use of service dogs, but not emotional support dogs, with participants over 45 years of age reporting lower perceived frequencies of misrepresentation of service dogs. A partial explanation for this perception can be found in prior research by Poulin [27], which found that levels of interpersonal trust increase as people age. However, the fact that this trend was only statistically significant for service dogs warrants further exploration. It is possible that longer history and stringent accreditation requirements associated with service dogs, specifically guide dogs [19], were determining factors in how these dogs are perceived. Additionally, the rather recent proliferation of ESAs in general [28] might have affected the public’s perception of emotional support dogs.

An interesting finding was that participants with friends/family who owned either an emotional support or a service dog felt that there was a higher proportion of fraudulent use of both types of assistance dogs. Since we did not ask participants to explain the rationale for their responses, we can only speculate about the cause for this finding. Further research is necessary to determine if this result is related to participants observing their friends/family engaging in fraudulent representation of an assistance animal. Another possible explanation could be greater awareness of the duties performed by a true service animal, and consideration of how many people actually need this level of support. Finally, participants may also be aware of how the increased prevalence of misrepresented animals delegitimizes their friend/family member’s need for their assistance animal.

Despite these statistical differences, in general, most respondents to our survey perceived relatively low rates of assistance dog misrepresentation. While the exact numbers vary by participants’ demographic characteristics, on the whole, most people feel that fewer than 25% of people with a service dog or emotional support dog are “taking advantage of the system” and fraudulently representing a pet as an assistance dog. Assuming that the results of this survey are representative of true public opinion, it appears that the media may be over-representing the views of a small, but vocal, minority when it comes to allegations of assistance dog fraud. This is not surprising, given recent research [29,30] and the old adage that “bad news sells”.

While the results of the survey describe a generally favorable landscape for assistance dogs, our findings must be interpreted within the limitations of this study. This study relied on participants to self-report their true opinions and knowledge regarding assistance dogs, thus there is the possibility for social desirability bias. However, this was mitigated as much as possible by anonymous data collection, and by tapping a pool of participants with no prior connections to the researchers. The age distribution of participants led to the classification of all subjects over 45 years of age into a single group. In order to more fully investigate the relationship between age and perceived rate of assistance dog representation, future research should involve a greater number of older adults, representative of a broader age range.

## 5. Conclusions

The use of animals is becoming more popular as the human-animal bond continues to strengthen and grow. Assistance animals are governed by a complex, and often overlapping, series of laws and regulations, which only helps to fuel confusion and lack of understanding of the tasks and work performed by each type. Having a more in-depth understanding of the perceptions and options held by a sample of adult members of the public regarding assistance dogs can help us to address the need for a standardization in language and definition for these types of animals. Although there has been much negative press surrounding the issue of misrepresentation of assistance animals, the true prevalence of behavior is likely lower than portrayed by the media, or at the very least, members of the general public perceive it to be lower. While the results of this study are limited by the sample size and online recruitment techniques employed, it begins to provide preliminary objective data on public perception of the roles played by different types of assistance dogs and pertinent rules and regulations. Even though prior research [31] shows that participants recruited through mTurk are more socioeconomically and culturally diverse than those recruited through other means, further research is needed to more fully explore opinions held by groups who were underrepresented in this survey. This includes older adults and individuals with limited English proficiency.

## Figures and Tables

**Table 1:** Participant demographics.

Participant Attribute	Frequency	Percent
**Gender**		
Male	135	47.5
Female	149	52.5
Total	284	100.0
**Age**		
18–25	42	14.8
26–35	131	46.1
36–45	64	22.5
Over 45	47	16.5
Total	284	100.0
**Highest Educational Level**		
High School	19	6.7
Some college	53	18.7
Four-year degree	97	34.2
Graduate degree	115	40.5
**Own a Pet Dog**		
No	131	46.1
Yes	153	53.9
**Friends/Family Own Service Dog**		
No	176	62.0
Yes	108	38.0
**Friends/Family Own Emotional Support Dog**		
No	163	57.4
Yes	109	38.4
NR (no response)	12	4.2

**Table 2:** Perceived confidence in defining various types of assistance animals.

Type of Assistance Dog	Very Comfortable	Somewhat Comfortable	Not Very Comfortable	Not at All Comfortable
**Service dog**	151 (53.2%)	97 (34.2%)	31 (10.9%)	5 (1.8%)
**Emotional support dog**	124 (43.7%)	102 (35.9%)	50 (17.6%)	8 (2.8%)
**Therapy dog**	88 (31.0%)	114 (40.1%)	66 (23.2%)	16 (5.6%)

**Table 3:** Attitudes towards assistance animals.

Survey Item	Strongly Agree	Somewhat Agree	Neither Agree nor Disagree	Somewhat Disagree	Strongly Disagree
I see nothing wrong with people having service dogs if they think they are useful	168 (59.2%)	76 (26.8%)	24 (8.5%)	15 (5.3%)	1 (0.4%)
I see nothing wrong with people having emotional support dogs if they think they are useful	146 (51.4%)	86 (30.3%)	33 (11.6%)	16 (5.6%)	3 (1.1%)
I see nothing wrong with providers using therapy dogs if they think they are helpful	160 (56.3%)	69 (24.3%)	34 (12.0%)	17 (6.0%)	4 (1.4%)
I am really not sure of the difference between service dogs and emotional support dogs and therapy dogs.	13 (4.6%)	47 (16.5%)	45 (15.8%)	89 (31.3%)	90 (31.7%)
I think only guide dogs for the blind should be given special privileges, but not other types of service dogs or emotional support dogs	22 (7.7%)	57 (20.1%)	48 (16.9%)	67 (23.6%)	90 (31.7%)

**Table 4:** Knowledge of legal enquiries pertaining to assistance dogs. Participants were asked to indicate which of the following questions they are legally permitted to ask in determining if an animal qualifies as an assistance dog.

Survey Item	Yes, I Can Legally Ask	No, I Cannot Legally Ask	Don’t Know
What is your disability?	75 (26.4%)	**138 (48.6%)**	71 (25.0%)
Is your dog a service dog that is required because of a disability?	**113 (39.8%)**	102 (35.9%)	69 (24.3%)
What task is your dog trained to perform?	**159 (56.0%)**	68 (23.9%)	57 (20.1%)
Can I see some proof of your disability?	45 (15.8%)	**163 (57.4%)**	76 (26.8%)
Can I see proof of your dog’s status (certification or ID card)?	127 (44.7%)	**81 (28.5%)**	76 (26.8%)

Note: Correct answers are highlighted in bold.

**Table 5:** Public perceptions of appropriate levels of access for assistance dogs. Participants were able to select as many types of assistance dogs as they felt applicable for each scenario.

Survey Item	Service Dogs	Emotional Support Dogs	Therapy Dogs	None	Don’t Know
I think landlords should be able to refuse these types of dogs.	43 (15.1%)	54 (19.0%)	64 (22.5%)	144 (50.7%)	31 (10.9%)
I think these types of dogs should be allowed to ride in airplane cabins.	172 (60.6%)	114 (40.1%)	115 (40.5%)	38 (13.4%)	35 (12.3%)
I think these types of dogs should be allowed to reside in school dorms.	170 (59.9%)	131 (46.1%)	112 (39.4%)	28 (9.9%)	38 (13.4%)
I think these types of dogs should be allowed in classroom settings.	163 (57.4%)	98 (34.5%)	108 (38.0%)	46 (16.2%)	36 (12.7%)

**Table 6:** Relationship of participants’ demographic characteristics with perceived prevalence of assistance animal misrepresentation, as assessed by the question “what percentage of people with a service dog/emotional support animal do you feel are just taking advantage of the system”.

Participant Attribute	Service Dogs					Emotional Support Dogs				
	0–5%	6–25%	26–50%	51–75%	76–100%	0–5%	6–25%	26–50%	51–75%	76–100%
**Gender**										
Male	47 (34.8%)	42 (31.1%)	31 (23%)	15 (11.1%)	0 (0%)	40 (29.6%)	45 (33.3%)	37 (27.4%)	11 (8.1%)	2 (1.5%)
Female	78 (52.3%)	34 (22.8%)	27 (18.1%)	7 (4.7%)	3 (2.0%)	57 (38.3%)	36 (24.2%)	27 (18.1%)	26 (17.4%)	3 (2.0%)
**Educational Level**										
High School	10 (52.6%)	5 (26.3%)	2 (10.5%)	2 (10.5%)	0 (0%)	8 (42.1%)	7 (36.8%)	3 (15.8%)	1 (5.3%)	0 (0%)
Some College	28 (52.8%)	16 (30.2%)	6 (11.3%)	2 (3.8%)	1 (1.9%)	23 (43.4%)	12 (22.6%)	9 (17.0%)	9 (17.0%)	0 (0%)
4 years degree	46 (47.4%)	27 (27.8%)	20 (20.6%)	4 (4.1%)	0 (0%)	32 (33.0%)	27 (27.8%)	26 (26.8%)	10 (10.3%)	2 (2.1%)
Graduate degree	41 (35.7%)	28 (24.3%)	30 (26.1%)	14 (12.2%)	2 (1.7%)	34 (29.6%)	35 (30.4%)	26 (22.6%)	17 (14.8%)	3 (2.6%)
**Age**										
18–25	16 (38.1%)	14 (33.3%)	7 (16.7%)	5 (11.9%)	0 (0%)	18 (42.9%)	8 (19.0%)	7 (16.7%)	8 (19.0%)	1 (2.4%)
26–35	43 (32.8%)	39 (29.8%)	34 (26.0%)	12 (9.2%)	3 (2.3%)	38 (29.0%)	38 (29.0%)	37 (28.2%)	17 (13.0)	1 (0.8%)
36–45	30 (46.9%)	19 (29.7%)	12 (18.8%)	3 (4.7%)	0 (0%)	20 (31.3%)	19 (29.7%)	18 (28.1%)	6 (9.4%)	1 (1.6%)
Over 45	36 (76.6%)	4 (8.5%)	5 (10.6%)	2 (4.3%)	0 (0%)	21 (44.7%)	16 (34.0%)	2 (4.3%)	6 (12.8%)	2 (4.3%)

**Table 7:** Relationship of participants’ exposure to assistance animals and pet ownership with perceived prevalence of service animal misrepresentation, as assessed by the question “what percentage of people with a service dog/emotional support animal do you feel are just taking advantage of the system”.

Participant Attribute	Service Dogs					Emotional Support Dogs				
	0–5%	6–25%	26–50%	51–75%	76–100%	0–5%	6–25%	26–50%	51–75%	76–100%
**Pet Dog Owners**										
No	63 (48.1%)	31 (23.7%)	23 (17.6%)	12 (9.2%)	2 (1.5%)	46 (35.1%)	42 (32.1%)	23 (17.6%)	16 (12.2%)	4 (3.1%)
Yes	62 (40.5%)	45 (29.4%)	35 (22.9%)	10 (6.5%)	1 (0.7%)	51 (33.3%)	39 (25.5%)	41 (26.8%)	21 (13.7%)	1 (0.7%)
**Friends/Family Own Service Dog**										
No	101 (57.4%)	40 (22.7%)	25 (14.2%)	8 (4.5%)	2 (1.1%)	67 (38.1%)	56 (31.8%)	33 (18.8%)	17 (9.7%)	3 (1.7%)
Yes	24 (22.2%)	36 (33.3%)	33 (30.6%)	14 (13.0%)	1 (0.9%)	30 (27.8%)	25 (23.1%)	31 (28.7%)	20 (18.5%)	2 (1.9%)
**Friends/Family Own Emotional Support Dog**										
No	93 (57.1%)	36 (22.1%)	22 (13.5%)	10 (6.1%)	2 (1.2%)	61 (37.4%)	56 (34.4%)	29 (17.8%)	13 (8.0%)	4 (2.5%)
Yes	27 (24.8%)	36 (33.0%)	34 (31.2%)	11 (10.1%)	1 (0.9%)	31 (28.4%)	22 (20.2%)	33 (30.3%)	22 (20.2%)	1 (0.9%)
